# Ten-Year Follow-Up of a Fragment Reattachment to an Anterior Tooth: A Conservative Approach

**DOI:** 10.1155/2017/2106245

**Published:** 2017-06-27

**Authors:** Luiz Mendes, Laisa Laxe, Leandro Passos

**Affiliations:** ^1^Department of Restorative Dentistry, Federal Fluminense University, Health Institute of Nova Friburgo, School of Dentistry, Rua Doutor Silvio Henrique Braune 22, 28625-650 Nova Friburgo, RJ, Brazil; ^2^Department of Restorative Dentistry, Federal University of Juiz de Fora, School of Dentistry, Rua Israel Pinheiro 2000, Bloco D9, Bairro Universitário, 35020-220 Governador Valadares, MG, Brazil; ^3^Department of Prosthetic Dentistry, Federal Fluminense University, Health Institute of Nova Friburgo, School of Dentistry, Rua Doutor Silvio Henrique Braune 22, 28625-650 Nova Friburgo, RJ, Brazil

## Abstract

This report describes the 10-year follow-up data of a patient who underwent fragment reattachment to the maxillary central incisor after coronal fracture with pulp exposure as well as the procedures followed for functional and esthetic adjustments. A 9-year-old female patient presented at the clinic of dentistry at the State University of Rio de Janeiro with a coronal fracture and pulp exposure of the right maxillary central incisor that had occurred immediately after an accident. The intact tooth fragment was recovered at the accident site and stored in milk. The treatment plan followed was to perform direct pulp capping and tooth fragment reattachment. When the patient was 14 years old, adhesion between fragment and remaining tooth was lost, and fragment reattachment was performed. Five years later, the same tooth presented clinical discoloration and absence of sensitivity during pulp vitality tests. Subsequently, a new treatment plan was formulated, which included endodontic treatment, followed by nonvital tooth bleaching and light-cured composite resin restoration. An esthetic and natural-looking restoration was achieved. Tooth fragment reattachment is not a temporary restorative technique and requires functional and esthetic adjustments over time to maintain the biomimetic characteristics of traumatized anterior teeth and predictable outcomes.

## 1. Introduction

Tooth trauma has been a common challenge for dental professionals because many different protocols for treatment are currently available. Coronal fractures of the anterior teeth often occur in children and adolescents, and the maxillary central incisors are the most often injured in accidents because of their vulnerable position in the mouth [1–3].

In several countries, the treatment of traumatized teeth has been greatly neglected. However, their treatment is very important for maintaining the quality of life of teenagers and for avoiding other problems arising from long-term trauma [1, 4].

Tooth avulsion and coronal fractures with or without pulp exposure often characterize the trauma of maxillary incisors. The type of treatment depends on the biological tissues involved in the trauma [3]. A conservative option of treatment for fractured incisors is reattachment of the tooth fragment when it is available. This technique offers some advantages over conventional prosthetic restorations [5]. These advantages include the maintenance of the color and shape of the original tooth, psychological benefits to the patients, the conservative nature of the treatment, and the speed and feasibility of the technique [6–8].

Furthermore, following the clinical and radiographic conditions of teeth with reattached fragment is crucial because some common pathologic changes may occur over time. These changes in injured teeth can include fragment detachment, discoloration, radiographic apical radiolucency, root resorption, and pulp canal obliteration [6, 8]. If any of these events occur, new clinical interventions may be planned and applied to recover the original esthetic and functional condition.

The lack of an esthetically satisfying result may impair the social interactions of school children and affect the self-confidence of young people [7, 9].

This case report aimed to describe the 10-year follow-up data of a patient who underwent tooth fragment reattachment to the maxillary central incisor after coronal fracture with pulp exposure as well as the procedures followed for functional and esthetic adjustments.

## 2. Case Presentation

**Figure 1:**
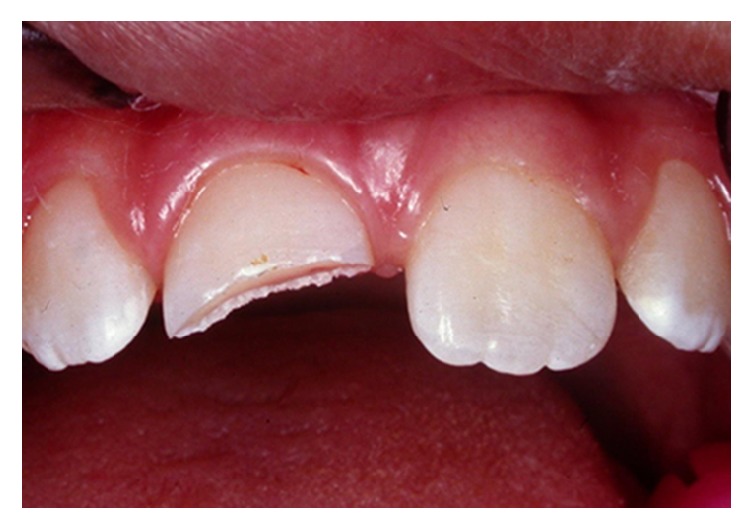
Clinical aspects of the crown fracture of the maxillary right central incisor.

**Figure 2:**
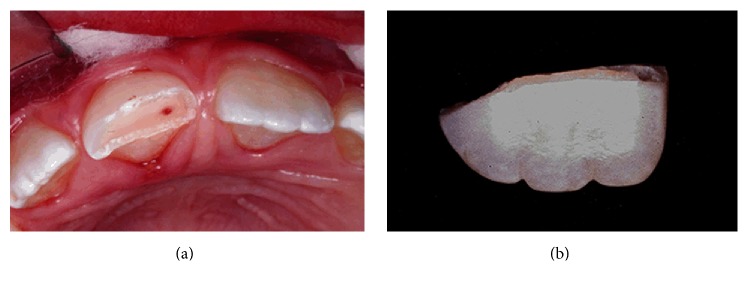
Clinical aspects of the pulp exposure (a). Original tooth fragment recovered at the accident site and stored in milk (b).

A 9-year-old female patient presented at the postgraduate clinic of dentistry at the State University of Rio de Janeiro, Rio de Janeiro, Brazil, with a coronal fracture and pulp exposure of the right maxillary central incisor (Figures 1 and 2) that had occurred immediately after an accident. On extra- and intraoral examination, no apparent trauma to the soft tissues was identified. The intact tooth fragment was recovered at the accident site, stored in milk, and brought to the clinic by her mother [10].

The traumatized tooth presented a small pulp exposure of approximately 1 mm. Periapical radiographic examination revealed complete root development, closed apices, no periapical pathology, and absence of root or alveolar bone fractures. Due to the tooth fragment being sufficiently hydrated, we devised a treatment plan that included performing direct pulp capping and original tooth fragment reattachment, which was accepted by the patient's parents.

A local anesthesia (Alphacaine 100; DFL, Rio de Janeiro, RJ, Brazil) was applied, and the affected tooth and adjacent teeth were isolated with a rubber dam (Madeitex, São José dos Campos, SP, Brazil). For direct pulp capping, the area was carefully irrigated with sterile saline and chlorhexidine 2.0% solutions (Chlorhexidine S; FGM, Joinville, SC, Brazil) and dried gently with sterile cotton pellets. The exposed pulp area was covered with calcium hydroxide paste (Biodinamica, Ibiporã, PR, Brazil), followed by self-hardening calcium hydroxide cement (Dycal; Dentsply Caulk, Milford, ME, USA) and light-cured glass ionomer cement (Vitrebond; 3M ESPE, St Paul, MN, USA) according to the manufacturer's recommendations.

The tooth fragment presented good positional stability (Figure 2). A dentinal groove (1.0–1.5 mm) was created inside the tooth fragment with a number 1012 diamond bur (KG Sorensen, Cotia, SP, Brazil) attached on a high-speed turbine (Kavo, Joinville, SC, Brazil) [11].

The tooth fragment and remaining dental structures were acid etched using 37% orthophosphoric acid (Ultra-Etch; Ultradent Products, Inc., SJ, UT, USA) for 30 s on the enamel margins and 15 s on the dentin substrate [12]. The acid was removed by rinsing with water, and the substrate was gently dried with cotton pellets. A conventional etch-and-rinse adhesive system (Scotchbond Multipurpose; 3M ESPE, St Paul, MN, USA) was applied on the conditioned enamel and dentin surfaces according to the manufacturer's recommendations [12].

**Figure 3:**
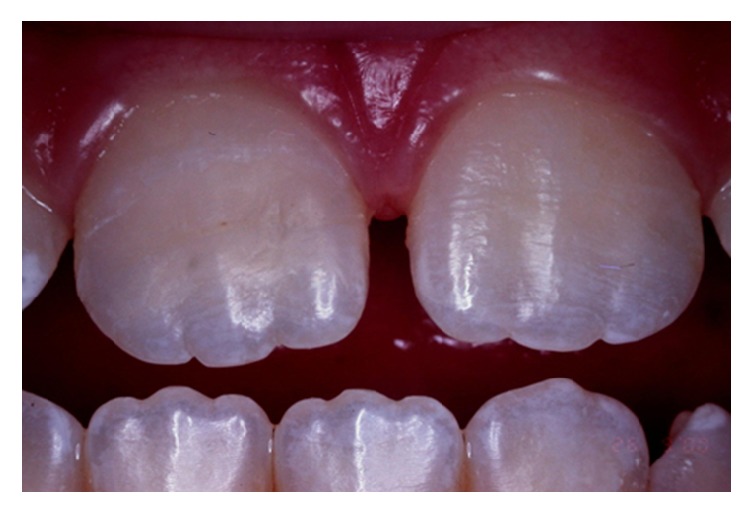
Clinical aspect of the original reattached tooth fragment.

Subsequently, the tooth fragment was reattached using dual composite resin cement (Variolink; Ivoclar Vivadent, Schaan, Liechtenstein). The residual excess cement was removed (Suprafill, SS White, Rio de Janeiro, RJ, Brazil) before light-curing the resin cement for 40 s on the buccal and palatal sides (Optilux 501; Demetron, Danbury, CT, USA). A good esthetic result was obtained, and beveling the adhesive interfaces was not necessary (Figure 3).

**Figure 4:**
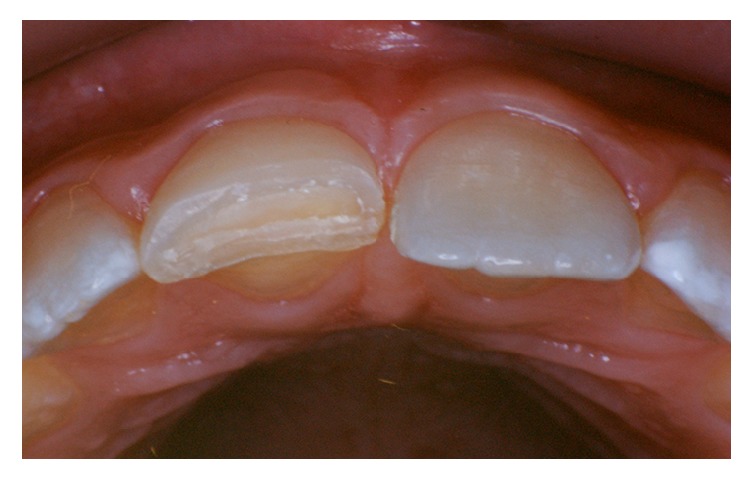
Tooth fragment detached after 5 years and development of the dentin barrier.

When the patient was 14 years old, the fragment detached and a new procedure for its reattachment was performed. The materials and protocol the same as those used during first reattachment procedure were followed, except for the direct pulp capping. The hard tissue barrier was complete and had remained intact after 5 years of protection (Figure 4) [6]. Clinical and radiographic examinations were performed every 6 months.

**Figure 5:**
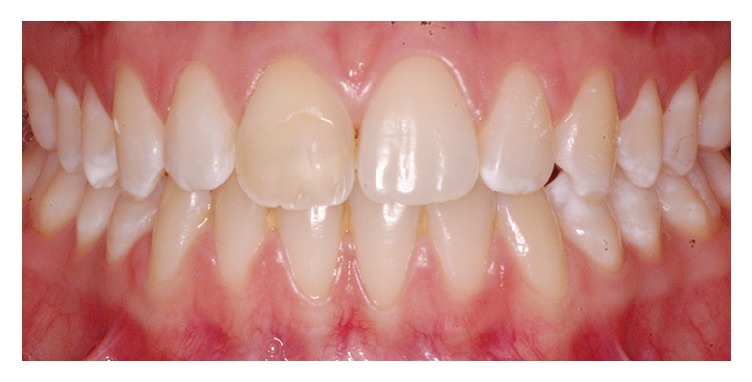
Discoloration of the fractured tooth crown after 10 years.

**Figure 6:**
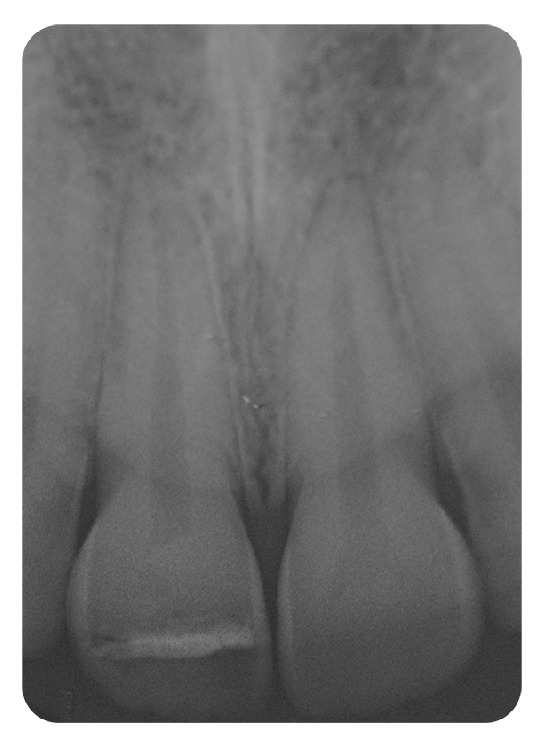
Radiograph showing periapical radiolucency.

**Figure 7:**
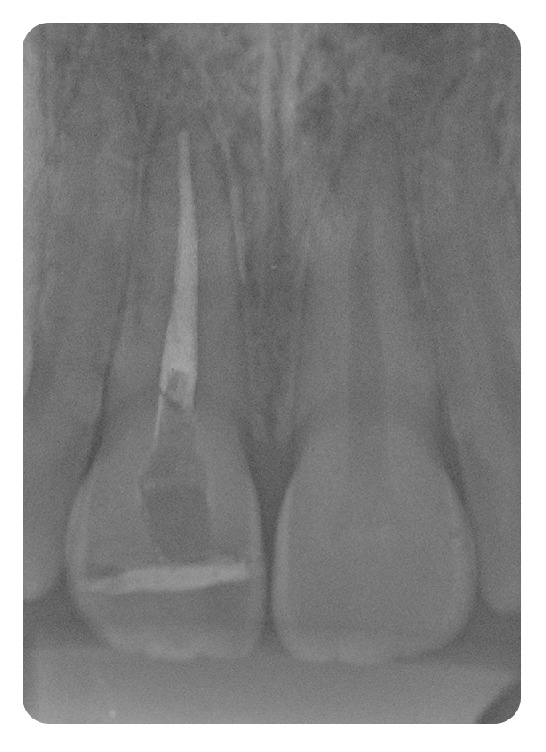
Radiograph after endodontic treatment.

During a follow-up 5 years later, the same right maxillary central incisor presented clinical discoloration (Figure 5) and absence of sensitivity during pulp vitality tests. A radiographic image revealed periapical radiolucency (Figure 6). The new treatment plan included endodontic treatment (Figure 7), followed by nonvital tooth bleaching and light-cured composite resin restoration.

Before initiating the endodontic treatment, an initial vinyl polysiloxane impression (Express, 3M ESPE, St. Paul, MN, USA) of the upper arch was obtained using a gypsum model (Durone IV, Dentsply, Brazil) to record the original anatomy of the teeth. A second impression was obtained using vinyl polysiloxane (Elite Transparent, Zhermack, Italy) for producing a transparent mask of the incisal and palatal surfaces. This transparent mask could act like a guide matrix during composite resin restoration after the endodontic treatment and dental bleaching.

**Figure 8:**
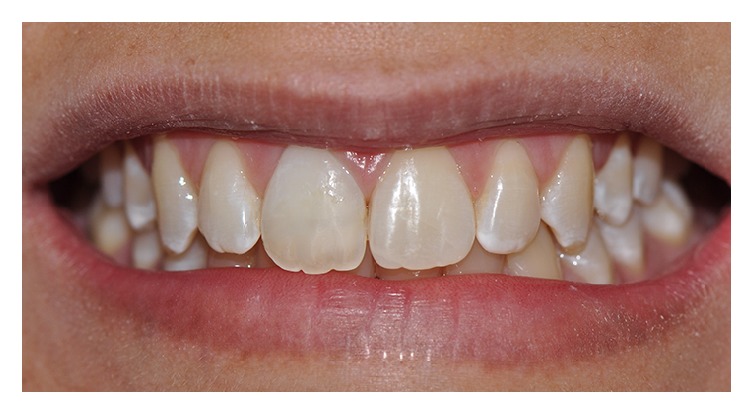
Clinical aspects after internal nonvital bleaching.

One week after ending the endodontic treatment, the injured tooth was isolated with a rubber dam and 3 mm root canal filling below the vestibular cement-enamel junction was removed. This area was covered with zinc phosphate cement to generate a mechanical barrier to the bleaching agents. Bleaching was performed with an intracanal paste of sodium perborate mixed with distilled water (Whiteness Perborato; FGM, Joinville, SC, Brazil), which was replaced every 5 days [13]. In total, the bleaching paste was replaced thrice. The final tooth color after bleaching is shown in Figure 8. An interval of 15 days after tooth bleaching was necessary before restoring the palatal access with light-cured composite resin.

The coronal access opening was restored with an etch-and-rinse adhesive system (Scotchbond Multipurpose; 3M ESPE, St Paul, MN, USA) and a light-cured nanofilled composite resin (Filtek Z350 XT, A2, 3M ESPE, St Paul, MN, USA) according to the manufacturer's recommendations. The incremental technique was followed, and each 2 mm layer of the composite resin was photopolymerized for 40 s (Elipar FreeLight 2, 3M ESPE, St. Paul, MN, USA). The final palatal surface was restored with a translucent composite resin (Pearl Neutral, Vitalescence, Ultradent Products, Inc., South Jordan, UT, USA) to replace the achromatic enamel. This last resin increment was positioned and photopolymerized on the palatal surface through the transparent silicon mask created before endodontic treatment.

**Figure 9:**
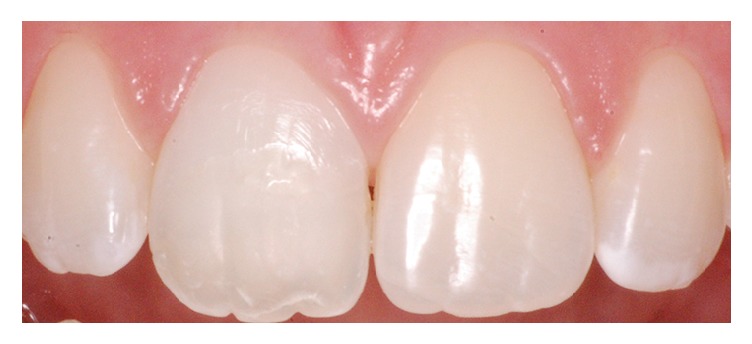
Clinical aspects after composite resin restoration.

Furthermore, esthetic recontouring with photoactivated composite resin was performed on the incisal third of the buccal surface to improve the biomimetic characteristics of the traumatized tooth after bleaching. The same restorative materials that were applied to restore palatal access were used (Figure 9).

The occlusion was checked by evaluation of the maximum habitual intercuspation and protrusive and lateral protrusive movements. The procedure was completed by performing occlusal adjustments using fine and extra-fine granulated diamond burs (KG Sorensen, Cotia, SP, Brazil).

Two weeks later, after rehydration of the tooth, final polishing was performed with polishing points (Jiffy; Ultradent Products Inc., South Jordan, UT, USA), followed by polishing paste (Enamelize; Heraeus Kulzer, Hanau, Germany) on a felt buff, and surface luster and shine were eventually obtained.

**Figure 10:**
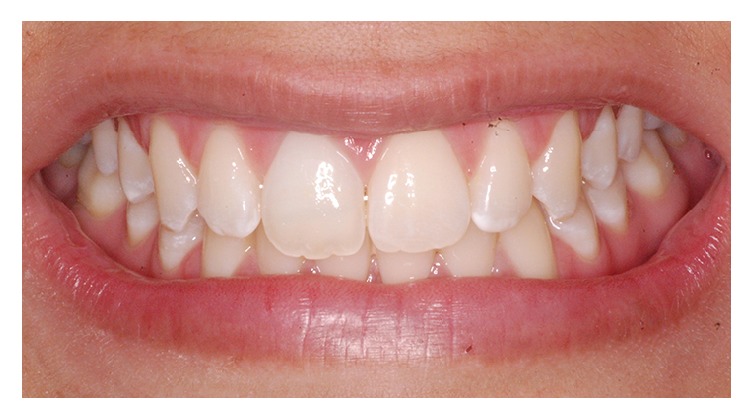
Final clinical appearance.

An esthetic and natural-looking restoration was achieved (Figure 10). This restoration completely satisfied the functional and esthetic expectations of the patient and dental team.

## 3. Discussion

Traumatic fractures of the anterior teeth are a common problem in children and adolescents because of their active lifestyle [3]. Proper dental treatment following a traumatic dental injury is crucial for preventing the biological and sociopsychological impacts [9].

Many techniques can be applied to restore fractured crowns, varying from original tooth fragment reattachment to full-coverage crown restorations. The treatment and prognosis for each case may differ according to the patient's age, amount of enamel available for bonding, wideness and wetness of the dentin tubules in young permanent teeth, possibility of bacterial contamination of the dentin and pulp, and availability of the tooth fragment for adhesion [12].

According to some studies, techniques for original tooth fragment reattachment offer an excellent treatment option for anterior fractured teeth because their original anatomic form, contour, color, surface texture, translucence, occlusal alignment, and function are maintained [3, 7, 14].

Numerous factors play an important role in determining how long the reattached tooth fragment remains functional. Among these factors, the media used to store the tooth fragment after fracture, type of material used for adhesion, use of materials to protect the dentin-pulp complex, flow of composite resins or cements, and technique used for the reattachment procedure are the most prominent [10, 12, 14]. However, recent studies have reported that different resin materials do not influence the fracture strength of the reattached tooth fragment [12, 14].

Some techniques of fragment reattachment include a bonding procedure without any type of wearing of the remaining tooth or tooth fragment surfaces. This technique is called simple reattachment [11]. However, some authors advocate wearing the tooth surfaces prior to or after bonding [6, 11]. The purpose of techniques like external chamfering or over contour and internal dentinal groove reattachment is to obtain optimal esthetics, retention, and function [6, 11, 14].

Limited data is available on the strength of reattached fractured fragments [11]. In this case, an internal groove was prepared in the tooth fragment surface. This groove had two objectives: (1) creating physical space for the material protecting the dentin-pulp complex and composite resin cement and (2) increasing the fracture strength of the reattached tooth fragment. Consequently, an accurate adaptation of the tooth fragment was achieved, and esthetic, functional, and biological parameters could be successfully maintained for 10 years [6, 8, 11].

Although some authors [3, 6] have reported the absence of clinical discolorations and pathological changes in the anterior teeth with an original fragment reattached, the follow-up duration of these cases was 24 and 12 months, respectively. This period can be considered short for evaluating the clinical success of some treatments, such as tooth fragment reattachment, in young people. In the present case, such changes were observed only 10 years after the first tooth fragment reattachment.

Thus, an immediate tooth fragment reattachment should not be considered as a temporary alternative treatment for fractured anterior teeth just because it offers an excellent restorative option for clinicians and patients [3, 8]. Fractured tooth fragment reattachment is not a final treatment [3] and may require new clinical interventions for esthetic, biological, and functional adjustments over time.

The esthetic appearance of the definitive restoration may cause a problem [9, 12]. Teeth may become discolored due to extrinsic or intrinsic factors. The main intrinsic factors are pulp hemorrhage, decomposition of the pulp, bacteria and their products, and pulp necrosis, among others. During physical trauma, blood vessels can be damaged, resulting in blood overflow into the pulp chamber. An intrinsic discoloration of the tooth crown can thus occur due to the diffusion of blood components into the dentinal tubules because of internal pulp bleeding. Over time, blood degradation products release iron during hemolysis, and degraded proteins of necrotic pulp tissue may also cause discoloration [15].

A more conservative treatment for discolored nonvital teeth is dental bleaching. The internal bleaching procedure is widely used because it is efficient, relatively simple, and inexpensive and preserves the dental hard tissue compared to the prosthetic treatment [16].

Sodium perborate-water mixture is the most suitable material used for internal bleaching because of the low extraradicular diffusion of hydrogen peroxide [13]. The amount of hydrogen peroxide penetration depends on the form of sodium perborate. The penetration is significantly higher with sodium perborate-hydrogen peroxide mixtures than with mixtures of sodium perborate tetrahydrate and water. Several studies have demonstrated that sodium perborate with water is an effective and safe bleaching agent [13, 17–19].

Finally, treatment in the anterior region is not considered a success when only the function and health of soft tissues and the remaining dental structures are recovered. The esthetic aspects of the restoration are equally important nowadays because of the high psychosocial and emotional impact on individuals' quality of life [9, 20].

Therefore, composite resin restoration is a conservative option to replace the palatal endodontic access. Composite layering is the key to obtaining esthetically successful restorations. This procedure is simplified by preparing a transparent silicon mask with vinyl polysiloxane based on a wax-up or on previous restorations or intact hard tissues with adequate form, as in this case [21, 22]. An appropriate silicone matrix is mandatory for the precise clinical reproduction of the ideal shape of the dentition [20].

In summary, treatment in the anterior region of the mouth after traumatic injuries requires satisfactory function, health of tissues, and great esthetic results [23]. Original tooth fragment reattachment can be considered the best option to recover fractured anterior teeth in patients younger than 18–20 years. The advantages presented by this conservative technique overcome any prosthetic treatment.

## 4. Conclusion

Techniques for tooth fragment reattachment are not temporary procedures but require functional and esthetic adjustments over time to maintain the biomimetic characteristics of traumatized anterior teeth with a very conservative approach and predictable outcomes. It should be considered when treating patients with coronal fractures of the anterior teeth, especially younger patients.
